# Factors Influencing Referral for Bariatric Surgery by Primary Care Physicians in Northern Israel

**DOI:** 10.1007/s11695-024-07253-x

**Published:** 2024-05-09

**Authors:** Elham Zoabi, Roni Elran-Barak, Nasser Sakran, Noga Kaftori Sandler, Ossama Abu Hatoum, Uri Kaplan

**Affiliations:** 1 School of Public Health, University of Haifa, 3103301 Haifa, Israel; 2 Department of General Surgery B, Emek Medical Center, Yitzhak Rabin Boulevard 21, 1834111 Afula, Israel; 3 Department of General Surgery, Holy Family Hospital, 1623409 Nazareth, Israel; 4 The Azrieli Faculty of Medicine Safed, Bar-Ilan University, 1311502 Ramat Gan, Israel; 5 Rappaport Faculty of Medicine, Technion-Israel Institute of Technology, 3109601 Haifa, Israel

**Keywords:** Primary care physician (PCP), Bariatric surgery, Referral

## Abstract

**Purpose:**

Obesity is a chronic metabolic disease with global distribution among adults and children which affects daily functioning and ultimately quality of life. Primary care physicians (PCPs) provide an important role for the treatment of severe obesity. Better understanding of obesity and its treatment options may increase patients’ referral rates to the various treatment modalities, including metabolic/bariatric surgery (MBS).

**Materials and Methods:**

A quantitative cross-sectional study used a self-reported questionnaire among PCPs of Clalit Health Services (CHS) in Northern Israel. The quantitative questionnaire examined the PCP’s knowledge, opinions, attitude, and approaches to managing severe obesity.

**Results:**

A total of 246 PCPs from Northern Israel filled the questionnaire (42.9%), the majority were Muslim Arabs (54.5%), who gained their medical degree outside of Israel (73.8%) and practicing for over 10 years (58.8%). 64.3% of PCPs had a high workload (over 100 appointments per week), 77.1% did not know the definition of severe obesity, and 69.17% did not attend educational meetings regarding obesity during the previous year. The referral rate for MBS was 50.4% ± 23.3. Two prognostic factors that had a statistically significant effect on the referral rate for bariatric surgery were the total appointments per week, and the number of practice years. Both had a negative association.

**Conclusion:**

The knowledge and referral rates for bariatric surgery are higher among PCPs with lower workload and relatively fewer practice years. Workshops and annual training courses may fortify knowledge and awareness for the treatment of obesity, which in turn could increase the referral rate for MBS.

**Supplementary Information:**

The online version contains supplementary material available at 10.1007/s11695-024-07253-x.

## Introduction

The worldwide prevalence of obesity has nearly tripled since 1975, in 2016, about 13% of the world’s adult population were obese [1]. Obesity is related to multiple comorbidities, including diabetes mellitus, cardiovascular disease, hypertension, hyperlipidemia, steatohepatitis, and cancer [2]. Weight loss has a major role in improving, and even remission, of obesity-related health conditions, as well as improvement in morbidity and mortality rates [3].

A survey held by the Israel Central Bureau of Statistics in 2017 determined that 48% of the population of Israel aged 20 and older were overweight or obese. Furthermore, within the population,46% of Israeli Jews and 54% of Israeli Arabs were overweight or obese [4]. However, the rate of metabolic/bariatric surgery (MBS) in Israel is gradually declining [5] while the Israeli population is increasingly becoming overweight or obese.

The most common measurement system for obesity is body mass index (BMI), which is defined by weight (in kilograms) divided by height (in meters) squared. The US Preventive Service Task Force (USPSTF) recommends all patients, who are 18 years or older, to be screened for obesity. Those with BMI above 30 kg/m^2^ (defined as obesity), or greater, should be offered additional interventions [6]. Therefore, the primary action in obesity management for patients is acknowledgment. Several treatment options for obesity are available for primary care physicians (PCPs), those may be a sole therapy or one of combined options. These therapies include behavioral-cognitive therapy, exercise, commercial weight loss programs, nutritional counseling, self-monitoring, pharmacological therapy, and MBS [6].

The World Health Organization has determined that MBS is an effective treatment for clinically severe obesity which significantly reduces mortality, associated illnesses, and significantly increases the life expectancy of patients. Currently, MBS is the most effective treatment option for clinically severe obesity and its related diseases [7, 8]. There are several bariatric surgical options which have been proven to be effective and safe. A patient’s decision regarding MBS is influenced by published medical evidence, local conditions, surgical trends, and PCP knowledge and experience [9]. Repeated encounters and close relationships with their patients provide the PCP with a major role in treating obesity and severe obesity.

Understanding the basic biology, pathophysiology of obesity and its effects on different body systems are the foundations for treating obesity [10]. Previous publications indicate that patients are more likely to attend weight loss programs which were advised by their PCP [11]. However, some PCP raised concern regarding their ability to influence patients in their weight loss effort [12]. A recent systemic review evaluating factors influencing PCPs referral for MBS found the following: post-operative complications, cost, availability, and adverse experiences resulted in lower referral rates, while patient request and motivation, and previous failed treatment for obesity and obesity-related comorbidities had a positive impact on PCPs referral rates for MBS [13].

Israel’s health care system is public and is subsidized by the government. Access to medical services is available and equal for all citizens. Clalit Health Services (CHS) is the largest of four health services found in Israel. It provides medical treatment based on insurance which subsidize medical treatment. Additional medical services can be purchased through private insurance companies. All PCPs that treat CHS patients are employed by CHS. CHS maintains weight loss program to their insured public. Indications for entering the program are according to the USPSTF recommendations.

The aim of this study is to examine the knowledge and attitude of CHS’s PCPs in Northern Israel regarding obesity treatment and MBS and to identify factors influencing referral for MBS.

## Methods

This is a questionnaire based, cross sectional study of CHS PCPs practicing family medicine in the Northern district of Israel. The study was performed between January 1, 2018, to November 30, 2018. In 2015, there were 573 PCPs practicing family medicine at the northern district of CHS [14], according to Israel’s Ministry of Health publication. The study was approved by CHS Review Board.

A referral protocol for MBS is implemented by CHS. A preliminary meeting with patients who expressed interest or were advised to undergo MBS is conducted by the PCP. They discuss obesity related comorbidities and the treatment option for severe obesity. If the patient meets the criteria for MBS, the PCP will refer the patient to a registered dietitian and a surgical consultation.

The following process validated the questionnaire. Based on current literature review regarding the topic of questionnaires to assess PCP knowledge, a preliminary questionnaire was developed by the bariatric center multi-disciplinary team at our institution. A group of ten experienced PCPs and three bariatric surgeons reviewed the questionnaire for the purpose of clarifying and modifying the questions. Thereafter, the questionnaire was presented to a pilot group of 30 PCPs for re-assessment regarding the clarity and suitability of the questions. The final questionnaire contained 29 questions (Appendix 1). The first part of the questionnaire consisted of questions regarding PCP demographics and their clinic characteristics. PCP workload was assessed by the total encounters per week with patients. The number of patients eligible for MBS, patients with severe obesity, was determined by multiplying the number of patients in the clinic by the percentage of severely obese patients in the clinic. The referral rate for MBS, specific to each PCP, was calculated by dividing the number of patients referred for surgical consultation by the number of patients eligible for MBS

1 $$\left(\mathrm{PCP}'\mathrm{s\;referral\;rate}=\frac{\mathrm{Patients\;referred\;for\;surgical\;consultation}}{\mathrm{Patients\;in\;PCP}'\mathrm{s\;clinic}\times\mathrm{Percentage\;of\;patients\;with\;severe\;obesity}}\right).$$

The second part of the questionnaire assessed the PCP’s knowledge and attitude toward the treatment of obesity and MBS.

Questionnaires were sent via e-mail to all PCPs working addresses, the email included a cover letter explaining the purpose of the study. Patients participating in the research did not receive financial compensation by filling out the questionnaires. The questionnaires itself did not contain identifying information, however, each PCP received a unique identifying number which assisted in sending two notifications emails, with a one-month interval in between, to all non-responders.

Analyses were performed using the IBM SPSS Statistical package (version 23.0, SPSS Inc, IBM Corp., Armonk, NY). Continuous variables were presented as means and standard deviations (SD), while categorical variables were presented as frequencies and percentages. Data normality was assessed using the Kolmogorov–Smirnov test. Between groups, differences were analyzed using the Kruskal–Wallis test, Chi-square test, or Fisher's exact test, as appropriate. All statistical tests were two-tailed, and statistical significance was set at *p* < 0.05.

## Results

### General

The questionnaire was filled out by 246 (42.9%) of CHS PCPs in Northern Israel. Table 1 summarizes the sociodemographic characteristics of PCPs who filled the questionnaire and their practice. The average age of the study population was 46.4 ± 12.3 years and 74.4% were male. The majority were Muslim Arabs (54.5%). Most of the PCPs had practiced for over 10 years (58.8%) and gained their medical degree outside of Israel (73.8%). The location of their practice was equally distributed between urban and rural areas (48.4% vs. 51.6%, respectively).

**Table 1:** Primary care physicians’ characteristics (*n* = 246)

Age (years) mean ± SD		46.4 ± 12.3
Female (%)		63 (25.6)
Religion	Jews (%)	59 (24.0)
	Christian Arabs (%)	40 (16.2)
	Muslims Arabs (%)	134 (54.5)
	Others (%)	13 (5.3)
Med. Uni. Country	Israel (%)	64 (26.2)
	Others (%)	180 (73.8)
Professional seniority	0–5y (%)	76 (31)
	6–10y (%)	25 (10.2)
	10–15y (%)	31 (12.7)
	> 15y (%)	113 (46.1)
Number of weekly appointments	< 50p/w (%)	24 (9.9)
	50–100p/w (%)	61 (25.3)
	101–150p/w (%)	59 (24.5)
	> 150p/w (%)	97 (40.3)
Practice type	Multi-disciplinary (%)	74 (30.8)
	primary care only (%)	166 (69.2)
Practice location	Urban (%)	117 (48.4)
	Rural (%)	125 (51.6)

*SD*, standard deviation; *y*, year; *p*, patients; *w*, week; *Med*. *Uni*., Medical university

According to the CHS database, the average patient appointments per week for a PCP is 125. In Northern Israel, 72% of CHS PCPs run a busy clinic with approximately 170 appointments per week and only 38% run a clinic which is considered less busy with around 70 appointments per week. Most of the PCPs in our study had a busy practice (64.3% had more than 100 appointments per week, of them 40.3% had more than 150 appointments per week).

### PCP Knowledge Regarding Obesity, Treatment of Obesity, and Bariatric Surgery

Most of the PCPs knew the definition of the term overweight (168(68.29%)), however, only 56(22.86%) knew the complete indications for MBS. The majority of PCPs (74.18%) agreed with the American Academy of Family Physician recommendation for overweight and obesity treatment. Nearly all PCPs sent their obese patients for dietitian consults (98.8%), while referral rates to join physical activity groups were lower (29.27%). The predominant reason for MBS referral was obesity-related comorbidities (29.04%), while patients’ refusal and psycho-social reasons (36.18% and 25.99%, respectively) were the main reasons for PCP’s not to refer severe obese patients to MBS. The percentage of morbidity and mortality following bariatric surgery (143 (59.8%) and 166(69.46%) respectively) was underestimated by most PCPs, however, only 40.17% will refer a family member who is classified as severe obese for surgical consult regarding MBS. Most of the PCPs did not attend an educational meeting regarding the treatment of obesity during the last year (69.17%).

### Predictive Factors for Bariatric Surgery Referral

The referral rate for bariatric surgery of severely obese patients in our study was 50.4% ± 23.3. As seen in Table 2, The only two predictive factors that reached statistical significance were the number of weekly appointments and the number of years in practice. Differences between MBS referral rates based on all categories of weekly encounters were statistically significant to *p* < 0.001. A post-hoc analysis found that differences exist between the lowest level of weekly encounters (< 50) and the highest level of the weekly encounter’s variable (> 150). PCPs with 50 or fewer appointments per week referred significantly higher rates of patients than PCPs with more than 150 encounters per week (62.31% vs. 43.92%, *p* = 0.004, respectively). Junior PCPs (less than 10 years in practice) referred significantly more patients for bariatric surgery than senior ones (53.44% vs. 48.28%, *p* = 0.048, respectively). Age, gender, ethnicity, location, and clinic type were not identified as a predictive factor for referral.

**Table 2:**
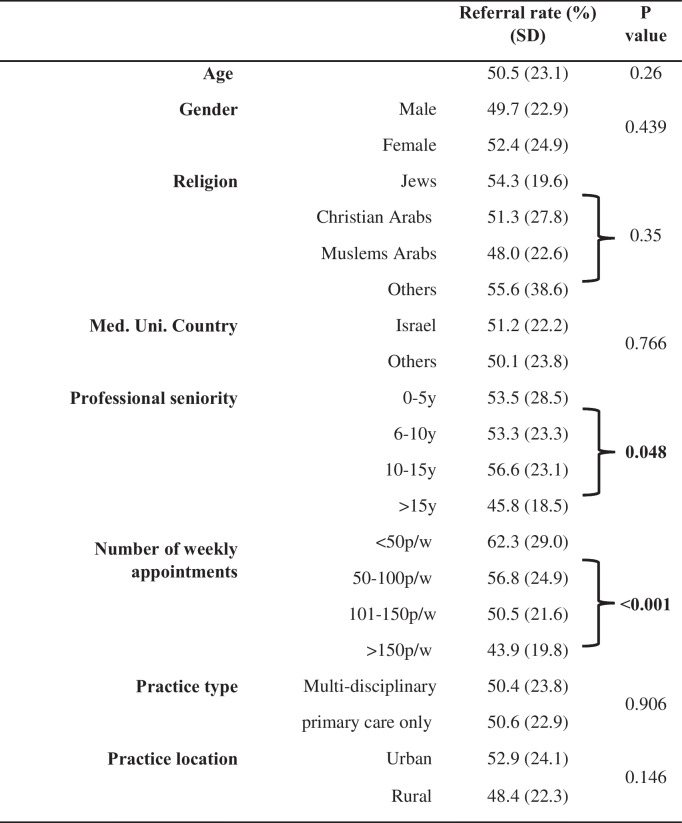
The relationship between demographics characteristics and referral rate for bariatric surgery

*SD*, standard deviation; *y*, year; *p*, patients; *w*, week; *Med*. *Uni*., Medical university

## Discussion

The purpose of this study was to examine the knowledge and attitude of PCPs in Northern Israel towards the treatment of obesity in general and then focus on identifying factors influencing PCPs in referring severe obese patient for MBS. We found that the general knowledge regarding obesity and MBS of CHS PCPs in Northern Israel was similar to the current literature [15]. An increased number of appointments per week and professional seniority were negative prognostic factors for referral to MBS among PCPs.

The shift from defining obesity as an individual fault to a chronic relapsing multifactorial and neurobehavioral disease had a major effect on the treatment of obesity. The practice guidelines on bariatric surgery were establish in 2004 and reestablished in 2020 [16]. However, the role of the PCP and possible intervention options are not clear [17] and our study found similar results. PCPs did not know the complete indications for MBS, which may be a possible reason for under referral for other treatment modalities besides MBS.

Previously reported studies highlighted that medical and surgical complication were the most common barrier PCPs had regarding bariatric surgery referral [13, 18]. In our study, although PCP underestimated the morbidity and mortality of MBS, they preferred not to refer a severe obese relative for bariatric surgery. These findings can indicate either the fear of the surgery or an under knowledge regarding the benefit of MBS. Most of the PCPs in our study did not attend an educational meeting during the last year regarding the treatment of obesity. Education and professional programs will improve the knowledge of PCPs and increase the referral for obesity treatment programs and MBS [13]. Advocating a nationwide obesity treatment program will lead to better advertisement of the different programs treating obesity. This in turn will lead to better treatment of the disease and the referral of a patient who understands the different options in treating obesity.

We identified two major factors influencing the referral rate for bariatric surgery. The number of years in professional seniority had a negative association with the referral rate, this phenomenon was described in previous studies [19]. Since 1945, the World Health Organization has recognized obesity as a disease, however, the American Medical Association recognized obesity as a disease only in 2013, classifying obesity as a new disease. This could be a possible reason for the higher referral rate of patients to undergo MBS by junior PCPs in comparison with older PCPs.

The relationship between workload and referral rate had conflicting results. While there are studies showing increased referral rate with increased workload [20, 21], others found opposite results [22]. Our study found a negative effect between workload, defined by number of appointments per week, and the referral rate for bariatric surgery. The treatment for obesity on the PCP level is a complex one. It allows the PCP to explore various treatment options with the patient [23]. Longer appointments can lead to better understanding of the disease by the patients, which in turn enable them to understand the treatment options including bariatric surgery.

One of the barriers in obesity management by the PCPs is their stigmatizing attitude towards patients with obesity. The negative stereotypes have implications on quality of care and health outcomes [24]. Insufficient medical training and education program causing PCPs to wrongly assume that obesity is mainly caused by personal behavioral factors rather than physiological and genetic causes labeling it as patient’s control disease [25]. Addressing stigmatizing behaviors during educational meetings could reduce this behavior and improve the treatment of obesity.

The current study highlighted factors influencing the treatment of obesity by PCPs. Constructing new educational programs in our region which address these factors along with a better understanding of the relationship between workload and causes for MBS referral will improve treatment of obesity in our region.

This study has a number of limitations. A response rate of 42.9% is a relatively high in terms of PCPs survey. However, the knowledge and behavior of the no responders could be different from the responding PCP to our survey. This was a self-reported survey; a recall bias could have influenced our result. In addition, our survey was a voluntary one, which may result in a self-selection bias as only PCPs interested in obesity could have chosen to answer the survey. Lastly, the work was performed only in the northern part of Israel and could not be applied to the rest of county due to possible socioeconomic differences.

## Conclusion

Our study provides important information regarding the factors influencing referral to MBS by PCPs of Northern Israel. Enhancing the understanding of both patients and PCPs, particularly among senior PCPs, while also reducing their workload, will facilitate informed discussions about treatment options. This, in turn, will enhance the management of obesity and increase the rate of referrals for MBS among severely obese patients. Conducting education and professional programs will further lead to a better understanding of the disease and the possible treatment options. Larger scale additional studies are needed to probe further into highlighting the factors associated with PCPs referrals for MBS.

### Supplementary Information

Below is the link to the electronic supplementary material.

Supplementary file1 (DOCX 30 KB)

## Data Availability

The data that support the findings of this study are available from the authors but restrictions apply to the availability of these data, which were used under license from Clalit Health Services for the current study, and so are not publicly available. Data are, however, available from the authors upon reasonable request and with permission from Clalit Health Services.

## Declarations

All procedures performed in studies involving human participants were in accordance with the ethical standards of the institutional research committee of Clalit Health Services and with the 1964 Helsinki declaration and its later amendments or comparable ethical standards.
