# Supplementary material for: Factors Influencing Referral for Bariatric Surgery by Primary Care Physicians in Northern Israel
Source: Obes Surg. 2024 May 9;34(7):2431–7. doi: 10.1007/s11695-024-07253-x (PMC11217136; doi:10.1007/s11695-024-07253-x)
Supplement: Supplementary file 1 — Supplementary file1 (DOCX 30 KB) [file 11695_2024_7253_MOESM1_ESM.docx]

Appendix1: PCP questionnaire

Here is a questionnaire that examines attitudes, awareness, and management of obesity treatment amongst family doctors.

The questionnaire is anonymous, and data will be used by the researcher, exclusively for the current study.

Are you willing to participate in this study and answer this questionnaire?

Yes / No

| **Part "A" Demographic Details** |
| --- |

| 1. Age: |  |
| --- | --- |
| 1. Sex: |  |
| 1. Origin: | 1. A Jew  2. Christian Arab  3. Muslim Arab  4. Other: _______ |
| 1. Years of seniority in family medicine: | 1. 0-5  2. 6-10  3. 10-15  4. Over 15 |
| 1. Medical degree obtained in (country): | 1. Israel  2. Abroad: ____ |
| 1. During the previous year, how many patients were registered at your clinic? | 1. Up to 100  2. 100-500  3. 500-1000  4. 1000-3000  5. Above 3000 |
| 1. Total number of weekly appointments? | 1. Less than 50  2. 50-100  3. 101-150  4. Above 150 |
| 1. Location of your clinic: | 1. City  2. Village |
| 1. Clinic classification: | 1. Independent  2. multi-professional |

| **Part "B" Treatment of Morbid Obesity** | |
| --- | --- |
| 1. How often do you weigh your patients? | 1. During every visit  2. Every six months  3. Once a year  4. More than a year  5. Not performed |
| 1. Do you use other tools other than weight to assess obesity? | 1. BMI  2. Waist circumference  3. Fat percentage test  4. Other: _________  5. No |
| 1. Have you attended education meeting for the treatment of obesity during the last year? | 1. Yes  2. No |
| 1. Overweight patients are considered in which BMI range? | 1. 25≤BMI<30 kg/m^2^  2. 30≤BMI<35 kg/ m^2^  3. 40≤BMI kg/m^2^ or 35≤BMI kg/m^2^ with a concomitant disease (ischemic heart disease, diabetes, obesity) |
| 1. In which BMI range is a patient defined as severely obese and should be considered for bariatric surgery? | 1. 25≤BMI<30 kg/^2^  2. BMI≥35 kg/m^2^  3. 40≤BMI kg/m^2^  4. 35≤ BMI<40 kg/m^2^ with accompanying disease (ischemic heart disease, diabetes, diabetes)  5. 40≤BMI kg/m^2^ or 35≤ BMI<40 kg/m^2^ with associated disease |
| 1. In the last year, what is the overall percentage of your patients who are severely obese? | 1. less than 5%  2. 5-10 %  2. 10-20%  3. 20-30%  4. Over 30% |
| 1. According to the AFP (American Family Physicians) guidelines regarding the treatment of overweight: patients defined as overweight should be referred for dietary and exercise counseling.    1. How many overweight patients did you refer for a dietitian consult during the last year? | 1. I did not refer  2. to 5  3. 6-10  4. 11-20  5. Over 20 |
| - 1. How many patients did you refer for physical activity during the last year? | 1. to 5  2. 6-10  3. 11-20  4. Over 20  5. I did not refer |
| 1. Do you recommend drug therapy to patients suffering from obesity? | 1. Yes  2. No |
| 1. If you answered yes to the previous question, with which medication did you have a positive experience? |  |
| 1. With which medication did you have a negative experience? |  |
| 1. According to the AFP guidelines regarding the treatment of excess weight: patients suffering from severe obesity should be sent for consultation to a surgeon who performs surgeries to treat severe obesity (bariatrics). Do you agree with the above statment? | 1. Yes  2. No |
| 1. Number of patients that you referred for bariatric surgery during the last year? | 1.0  2. 1-5  3. 6-10  4. 11-15  5. 16-20  6. Over 20 |
| 1. Number of patients in your clinic who underwent bariatric surgery during the last year? | 1.0  2. 1-5  3. 6-10  4. 11-15  5. 16-20  6. Over 20 |
| 1. Of your severely obese patients, what percentage request bariatric surgery? | 1. Up to 10%  2. 10-20%  3. 20-30%  4. 30-40%  5. Over 40% |
| 1. If you choose not to send a patient to undergo bariatric surgery, which is the most common reason for your dismissal? (Please mark the most correct answer) | 1. There are not enough resources at my disposal  2. Lack of awareness about the surgery or its benefits  3. Psychosocial considerations among patients  4. Insignificant results after surgery  5. Feeling discomfort in the continuation of the management of the treatment after the operation  6. Refusal of the patient |
| 1. Which is the most common cause for you to refer a patient for surgical consult regarding bariatric surgery? | 1. Failure in diet and exercise  2. Failure of drug treatment  3. Associated background diseases  4. BMI range  5. Success among other patients |
| 1. Which bariatric surgery do you recommend for a patient suffering from severe obesity (BMI=42) without underlying diseases? | 1. Sleeve gastrectomy  2. Roux-en-Y gastric bypass  3. Adjustable gastric band  4. Duodenal switch/ biliopancreatic diversion  5. Mini Gastric Bypass |
| 1. Which bariatric surgery do you recommend for a patient suffering from severe obesity (BMI=42) and diabetes mellitus? | 1. Sleeve gastrectomy  2. Roux-en-Y gastric bypass  3. Adjustable gastric band  4. Duodenal switch/ biliopancreatic diversion  5. Mini Gastric Bypass |
| 1. Out of the following bariatric surgeries, which do you believe is the most successful? | 1. Sleeve gastrectomy  2. Roux-en-Y gastric bypass  3. Adjustable gastric band  4. Duodenal switch/ biliopancreatic diversion  5. Mini Gastric Bypass |
| 1. What is the morbidity rate within 30 days of bariatric surgery? | 1. 1-5%  2. 5-10%  3. 10-15%  4. Above 15% |
| 1. What is the mortality rate within 30 days of bariatric surgery? | 1. 0.05-0.1%  2. 0.1-0.5%  3. 0.5-0.8%  4. Above 0.8% |

| Please indicate how much you agree/disagree with the following statements  (1- do not agree at all, 7 - completely agree) | | | | | | | |  |
| --- | --- | --- | --- | --- | --- | --- | --- | --- |
|  | 1. Patients suffering from severe obesity should try lifestyle modifications and dietitian consult before they are referred for surgical consult regarding bariatric surgery. | | | | | | | |
|  | 7 | 6 | 5 | 4 | 3 | 2 | 1 | |
|  | 2. I know patients suffering from severe obesity who lost weight without surgery. | | | | | | | |
|  | 7 | 6 | 5 | 4 | 3 | 2 | 1 | |
|  | 3. I will refer a severely obese family member for bariatric surgery. | | | | | | | |
|  | 7 | 6 | 5 | 4 | 3 | 2 | 1 | |
|  | 4. I feel that I can treat and monitor my bariatric surgery patient. | | | | | | | |
|  | 7 | 6 | 5 | 4 | 3 | 2 | 1 | |
|  | 5 . I feel comfortable referring severely obese patients for bariatric surgery after conservative/pharmacological therapy has failed. | | | | | | | |
|  | 7 | 6 | 5 | 4 | 3 | 2 | 1 | |
